# GiNA, an Efficient and High-Throughput Software for Horticultural Phenotyping

**DOI:** 10.1371/journal.pone.0160439

**Published:** 2016-08-16

**Authors:** Luis Diaz-Garcia, Giovanny Covarrubias-Pazaran, Brandon Schlautman, Juan Zalapa

**Affiliations:** 1 University of Wisconsin, Department of Horticulture, Madison, Wisconsin, United States of America; 2 Instituto Nacional de Investigaciones Forestales y Agrícolas y Pecuarias, Aguascalientes, Mexico; 3 USDA-ARS, Vegetable Crops Research Unit, University of Wisconsin, Madison, Wisconsin, United States of America; Beijing Forestry University, CHINA

## Abstract

Traditional methods for trait phenotyping have been a bottleneck for research in many crop species due to their intensive labor, high cost, complex implementation, lack of reproducibility and propensity to subjective bias. Recently, multiple high-throughput phenotyping platforms have been developed, but most of them are expensive, species-dependent, complex to use, and available only for major crops. To overcome such limitations, we present the open-source software GiNA, which is a simple and free tool for measuring horticultural traits such as shape- and color-related parameters of fruits, vegetables, and seeds. GiNA is multiplatform software available in both R and MATLAB^®^ programming languages and uses conventional images from digital cameras with minimal requirements. It can process up to 11 different horticultural morphological traits such as length, width, two-dimensional area, volume, projected skin, surface area, RGB color, among other parameters. Different validation tests produced highly consistent results under different lighting conditions and camera setups making GiNA a very reliable platform for high-throughput phenotyping. In addition, five-fold cross validation between manually generated and GiNA measurements for length and width in cranberry fruits were 0.97 and 0.92. In addition, the same strategy yielded prediction accuracies above 0.83 for color estimates produced from images of cranberries analyzed with GiNA compared to total anthocyanin content (TAcy) of the same fruits measured with the standard methodology of the industry. Our platform provides a scalable, easy-to-use and affordable tool for massive acquisition of phenotypic data of fruits, seeds, and vegetables.

## Introduction

The last decades have been marked by important biological discoveries driven in large part by the acquisition of massive amounts of genomic data. To fully utilize this knowledge and to apply it to plant breeding programs and the agricultural industry, strategies focused on quantifying phenotypic traits must evolve as quickly as the genome sequencing technologies [1]. Most of the current tools for high-throughput phenotyping in crop genetics research or industry are species-dependent and very complex to implement [2–6]. In most cases, the phenotyping technology is not accessible for small research groups or scientists working in minor crops.

Traditionally, breeding programs of minor crops have focused on a limited set of traits such as yield, fruit quality and resistance to abiotic or biotic stress using manually-collected phenotypic data. Manual phenotyping approaches are limited by the amount of data that can be recorded and processed, by the human error intrinsic to the measurement process, and by the difficulty of quantifying complex traits such as fruit or seed color [7]. Therefore, the availability of new technologies for massive acquisition and analysis of phenotypic data is critical to develop newer, more efficient strategies for genetic improvement in minor crops.

Machine vision technology has been successfully applied in different agricultural sectors for yield prediction [8,9], measuring physiological status of the plant in response to stresses [10], precision farming, fruit sorting and classification [11,12], and automated phenotyping [13]. Using automatic imaging technology allows the acquisition of quantitative data accurately, consistently, and nondestructively [13]. Some of these automatic systems for image data collection have already been used in quantitative trait loci (QTL) mapping and genome wide association studies. For example, image-based technologies and genetic association analysis were used in rice in order to identify the architecture of temporal salinity response [4]. The use of a rice automated and scalable phenotyping system allowed the collection of data for association analysis based on 97 digital traits in almost 400 genotypes over a 14-day period. In *Arabidopsis thaliana*, genome-wide association mapping of growth was conducted by combining top-view imaging, high-throughput image analysis, modeling of growth dynamics, and end-point fresh weight determination [14]. In triticale, a non-invasive and highly precise methodology was applied to identify QTLs related with the dynamic temporal patterns of biomass accumulation [3]. The previous examples are a small sample of studies in which image-based phenomics has been applied to measure important plant traits and to conduct genome-wide and QTL studies.

For the most part, phenomics has not been fully exploited in fruit crop research. Fruit phenomics studies have predominantly focused on developing detection, sorting and classification systems for the commercial fruit industry. Rakun *et al*. [15] created a system for apple yield prediction by detecting fruits in orchard trees using spatial-frequency based texture analysis and multi-view geometry. In citrus, a multivariate image-based approach was developed for automatically detecting skin defects [16]. Also, a very straightforward method based on neural networks for determining cherry color parameters during ripening was developed by Taghadomi-Sabery *et al*. [17]. Gonzalo and van der Knaap [18] used Tomato Analyzer for the genetic analysis of tomato varieties that exhibited elongated fruit shape.

In this study, we present a simple, user friendly, and scalable software called GiNA for the accurate measurement of shape and color related traits in fruits, vegetables and seeds. GiNA is a multiplatform software available in R and MATLAB^®^ that uses conventional pictures to perform high-throughput measurements of the physical properties of different objects. We emphasize the utility and applicability of GiNA for minor crops such as horticultural crops, where phenotyping resources are limited.

## GiNA, step by step

### An overall description of the platform

The GiNA platform was designed to solve the common deficiencies of traditional phenotyping methods such as reproducibility, scalability, and affordability. Nowadays, several phenotyping systems are available, but most of them are very complex to use, are not robust enough for high-throughput phenotyping, are too expensive, or work only for the plant species for which they were created. GiNA’s platform uses a very simple strategy for measuring shape and color trait parameters and offers several advantages: 1) it is highly reproducible, 2) it does not require programing experience, 3) it can be applied to many different crops, and 4) the cost of implementation and equipment required can be as low as $300 USD.

**Fig 1:**
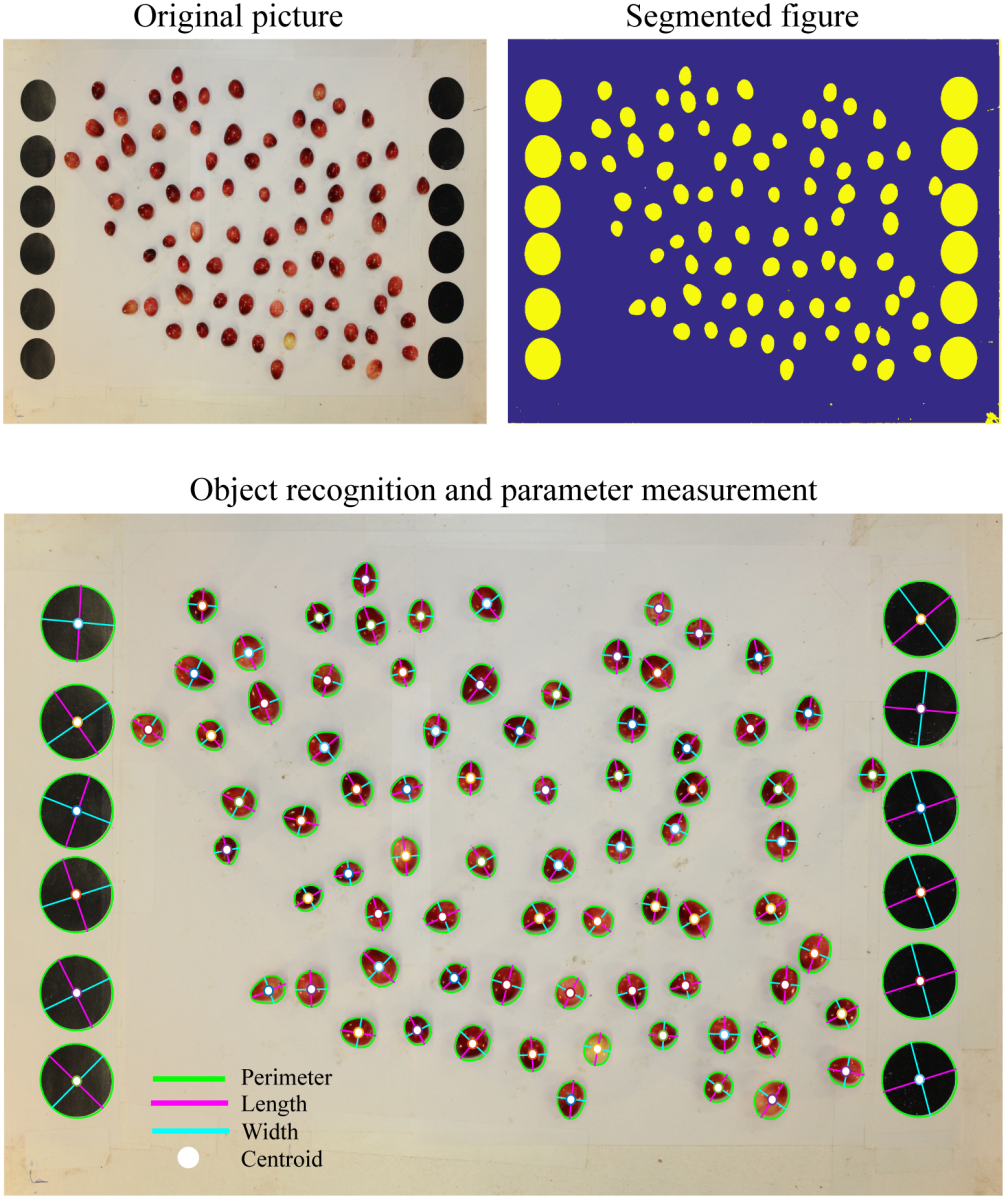
Example of computations performed by GiNA for background extraction on cranberry fruits. The algorithm works in two steps, image segmentation (by applying a predefined threshold or using a neural network approach) and object recognition to calculate the physical parameters.

GiNA relies on two main steps, conventional digital-picture recording and image processing by either R or MATLAB^®^ (users’ choice) algorithm implementation (Fig 1). A brief description of the software is provided in this manuscript; for more details and operating instructions, an illustrative tutorial is available at: http://cggl.horticulture.wisc.edu/home-page/ and in The Comprehensive R Archive Network (https://cran.r-project.org).

### Picture recording

Our methodology can measure fruits, vegetables and seeds of different sizes, from millimeters (such as peas) to decimeters (such as melon or squash fruit). Although we have included features in GiNA to help cope with image to image variation, parameter standardization for each project and across photography sessions must be performed to ensure best results. For example, GiNA’s users must standardize lighting conditions and camera settings such as light sensitivity, shutter speed, aperture and avoid camera flashes to reduce light reflection, which can appear as bright spots on objects in the image. In addition, camera alignment with the center of the background and proper focus on the fruits is required for best results. Since GiNA precisely estimates different parameters related with the shape of the object, the users must arrange the fruits/seeds in the scene such that they do not touch. Finally, references on each side of the background must be included to allow data normalization. The pictures analyzed in this study were taken using a home-made camera holder (as the shown in S1 Fig) and a DSLR camera Canon EOS REBEL T5. However, simpler cameras and/or tripod setups can be used as long as the described requirements are met.

### Algorithm implementation

The image-processing algorithm implemented in GiNA is divided into two parts, image segmentation and object analysis. The image-segmentation step performs a background extraction and prepares the image for data collection in the next step. The data-analysis step uses the segmented image and applies built-in R or MATLAB^®^ functions for extracting object properties. Finally, the measurements of all objects in the image are grouped and normalized for their comprehensive analysis.

Depending on the color contrast between the object (e.g., fruit) and background, two strategies can be applied for segmentation. When there is a very marked contrast between the object and the picture background, a threshold in one or multiple RGB channels can be set as cutoff value for removing the background from the picture. Commonly, darker (on a white background) or lighter (on a dark background) objects with small color variation can be analyzed by using threshold-based segmentation. When there is significant variation in the color of the object (e.g., one side of the object is dark red and the other side is yellow), or there is not evident contrast between the objects and background, a neural network approach can be implemented. This strategy requires an extra set of images including one picture of the color and texture variation in the background (without the references), and another one from the objects (see S2 Fig for examples of training pictures in cranberry). These figures can easily be made by creating a collage of screenshots. These two figures (or more if multiple colors are present in the fruits/vegetables) are used for training the neural network. GiNA automatically reads (in RGB format) each of the images provided, extracts the R, G and B values of each pixel and uses them as input for generate the network. Subsequently, GiNA produces 2 numeric values (or more if multiple figures representing the fruit color were provided) corresponding to the probability of each pixel to be part of the background or the fruits/vegetables. In terms of the network architecture, it consists of 10 hidden neurons, 70% of the pixels are used for training, 15% for validation, and 15% for testing. Data division is assigned randomly, and network training is done using scaled conjugate gradient and the performance is evaluated minimizing cross-entropy. Increasing resolution of the training pictures provides increased network performance for classifying pixels, but pictures of 1000x1000 pixels are sufficient for most analyses. The neural network approach can be also used in all the same situations that the threshold-based segmentation strategy is appropriate.

Once the segmentation has been applied (either by the threshold-based or neuronal network approach), it is necessary to clean the segmented figure in order to optimize the measurement computation. For example, if too much light was used in the picture, the inner parts of the fruits will be recognized as background. To deal with this situation, GiNA uses an algorithm for filling in holes in each picture. In other cases, and depending of the camera and lens used, the picture can have less illumination around the borders (even when external sources of light are used); in this situations, GiNA uses a function to stabilize the illumination among all the areas. Pictures corrections such as light border adjustments and filling in holes are applied automatically, thus the user does not have to take any additional processing steps.

Following segmentation of all the objects and proper post processing, customized functions normalize the photos using the references and generate biologically useful measurements for all objects. Among the measurements that this platform can obtain are those related to the object’s shape (e.g., two-dimensional area, length, width, shape, projected skin surface, two-dimensional perimeter, projected volume, eccentricity and solidity). Additionally, color related measurements such as RGB color, gray-scale color and color variation are also computed. All these parameters are computed solely based on the segmentation mask. A better description mathematical definition of the parameters generated by GiNA is available in S1 Table.

For user practicality, GiNA was written as a single function for both R and MATLAB^®^. However, multiple arguments can be declared to obtain the best estimates from the pictures, such as (1) the directory location of the pictures to be analyzed, (2) a resizing factor to reduce the number of pixels (large pictures take more time to process), (3) the minimum area of an object to be considered, (4) the desired segmentation method (either threshold or neural network approach), (5) the RGB channels to be used if the threshold segmentation method is used, (6) the number of references in each picture, (7) the actual size of the references in a specific unit (e.g., cm or inches) in order to convert the corresponding measurements (originally produced in pixels), and (8) arguments to generate figures and write.*csv* files of the data.

### GiNA produces consistent data under different system configurations

The GiNA software is very robust and produces consistent data even in variable lighting conditions and with different camera setups. A common concern about phenotyping systems based on conventional imaging a threshold-based segmentation methods is that the pictures must have extremely standardized lighting conditions and that the objects must be arranged in a specific way. In order to test GiNA’s robustness to cope with unfavorable photographic conditions, we performed an analysis to measure the correlation between measurements under different lighting conditions and different camera setups.

Table 1 shows the correlation between three pictures (containing the same 24 fruits) under different lighting conditions. In order to simulate light variations, different shutter speeds in the camera were used. As illustrated in S3 Fig, the pictures used are extreme cases of light variations. Among all the variables, a correlation of 0.92 was obtained, but most correlations were above 0.98 for all the comparisons between shape-related parameters, except for solidity (the extent that the shape is convex or concave), which had low correlation values among different lighting conditions. As expected, the parameters related with color showed lower average correlation (gray-scale color = 0.94 and color variation = 0.83) than shape and size related parameters. In general, although our tests showed that GiNA can cope with variable lighting conditions, we recommend to implementing a light standardization procedure prior to the start of massive phenotyping and maintaining the same lighting conditions throughout the experiment.

**Table 1:** Pearson’s correlation between three pictures taken using different lighting conditions. Shutter speed was modified in each picture in order to simulate light variation. All correlations were statistically significant at *p-value*<0.05. Used for this test can be found in S3 Fig.

Parameters		Lighting conditions		
		Normal (N)	Overexposed (O)	Dark (D)
ISO		400	400	400
Shutter speed		80	20	400
Segmentation channel		Blue	Blue	Blue
Threshold value		50	125	10
Shape	N	1	0.88	0.92
	O	0.88	1	0.97
	D	0.92	0.97	1
Length	N	1	0.98	0.98
	O	0.98	1	1
	D	0.98	1	1
Width	N	1	0.98	0.98
	O	0.98	1	1
	D	0.98	1	1
Area	N	1	0.99	0.99
	O	0.99	1	1
	D	0.99	1	1
Perimeter	N	1	0.98	0.99
	O	0.98	1	1
	D	0.99	1	1
surface	N	1	0.99	0.99
	O	0.99	1	1
	D	0.99	1	1
Volume	N	1	1	1
	O	1	1	1
	D	1	1	1
Eccentricity	N	1	0.86	0.90
	O	0.86	1	0.96
	D	0.90	0.96	1
Solidity	N	1	0.15	0.40
	O	0.15	1	0.51
	D	0.40	0.51	1
Gray-scale color	N	1	0.94	0.93
	O	0.94	1	0.97
	D	0.93	0.97	1
Color variation	N	1	0.90	0.83
	O	0.90	1	0.77
	D	0.83	0.77	1

In addition to testing GiNA’s performance under different lighting conditions, we evaluated the consistency of the data produced when changing camera positions. To do that, we photographed cucumber seeds at different camera heights (7, 15 and 22 inches from the background) and obtained Pearson’s correlation between measurements (see the pictures used in this test in S4 Fig). Table 2 shows an average correlation = 0.79 among all camera setups. Most of the shape-related parameters showed high correlation; however, because the flat shape of the seeds volume and solidity showed lower correlations than some of the other traits with different heights of the camera. Additionally, due to the decreased number of pixels in the measured objects with increased camera heights, gray-scale color and color variation possessed comparatively low average correlations in our height tests, 0.22 and 0.51, respectively. Although our tests indicated that different height camera setups can produce bias, we suggest that the bias can be easily avoided by using the same camera settings during each experiment.

**Table 2:** Pearson’s correlation between three pictures on different system setups. All pictures contained the same 25 seeds and were taken at the same focal distance and all other camera parameters were the constant. Pictures used for this test can be found in S4 Fig.

Parameters		Camera location (inches from background)		
		7 (L1)	15 (L2)	22 (L3)
Segmentation channel		Blue	Blue	Blue
Threshold value		95	100	105
Shape	L1	1	0.91	0.92
	L2	0.91	1	0.99
	L3	0.92	0.99	1
Length	L1	1	0.97	0.98
	L2	0.97	1	0.99
	L3	0.98	0.99	1
Width	L1	1	0.60	0.73
	L2	0.60	1	0.97
	L3	0.73	0.97	1
Area	L1	1	0.75	0.85
	L2	0.75	1	0.98
	L3	0.85	0.98	1.00
Perimeter	L1	1	0.93	0.89
	L2	0.93	1	0.91
	L3	0.89	0.91	1
Surface	L1	1	0.85	0.91
	L2	0.85	1	0.99
	L3	0.91	0.99	1
Volume	L1	1	0.63	0.76
	L2	0.63	1	0.97
	L3	0.76	0.97	1
Eccentricity	L1	1	0.95	0.95
	L2	0.95	1	0.99
	L3	0.95	0.99	1.00
Solidity	L1	1	0.84	0.26
	L2	0.84	1	0.36
	L3	0.26	0.36	1
Gray-scale color	L1	1	-0.18	0.33
	L2	-0.18	1	0.51
	L3	0.33	0.51	1
Color variation	L1	1	0.42	0.39
	L2	0.42	1	0.73
	L3	0.39	0.73	1

In conclusion, our test showed that GiNA is robust enough to deal with moderate-to-strong variations in lighting conditions. However, lighting conditions are technically harder to control and maintain constant among several photography sessions. On the other hand, our software produced less consistent data when different camera heights were tested for some of the examined traits. Nevertheless, camera height and other camera settings are simple and easy to control with proper camera setup and equipment use. Given the difficulty controlling lighting conditions, we suggest that the careful standardization of light is a prerequisite for using GiNA in most experiments as is the case when using other similar software for horticultural phenotyping [17, 18].

### Validation of GiNA with manually-taken measurements

The validation of GiNA was performed separately for color related measurements and shape-related measurements. For color measurements, total anthocyanin content (TAcy) in cranberry fruits was correlated with the color estimation produced by GiNA from images of the same fruits. Over a 4-week period, 22 samples of cranberry fruits from multiple cultivars were collected from a commercial cranberry farm. The field component of this study was performed at Cranberry Creek Cranberries Inc., Wisconsin USA with permission of Bill Hatch and Nicole Hansen. None of the field studies conducted involved endangered or protected species. Sampling consisted of picking all fruits in one square foot ring at three different locations in the same field. The fruits from the three locations were mixed, and a random selection of 100 fruits were used for taking pictures in four batches of 25 fruits. Then, the fruits were mixed again with the rest of the sample and TAcy was determined according to Vorsa *et al*. [19]. The fruits were harvested during the cranberry ripening period. During this time, fruit color changed from yellow to red, which provided a wide color spectrum for the validation analysis. Since anthocyanins are accumulated primarily in the fruit skin [20], and therefore responsible of fruit coloration in cranberry, high correlation between the two measurements was expected.

GiNA’s algorithm is very robust in terms of copping with different picture conditions (e.g., the presence of lights or shadows) when used for measuring shape-oriented parameters. However, color-related parameters can be affected by variations in natural light if pictures are taken on different days. Since cranberry pictures were obtained multiple times over a 4-week period, slightly differences in light were detected even when the camera parameters were not changed. Color validation was made by bootstrapping cross-validation (five-fold), in which 82% of the data points (TAcy measurements and digital color estimates, selected at random, with replace) were used for build a model *Y*_*ij*_ ~ *DC*_*i*_ + *Date*_*j*_, where *Y*_*ij*_ is the TAcy of the *i* sample in the *j* day, *DC*_*i*_ is the digital color of the sample *i*, and *Date*_*j*_ is the date *j* when the picture was taken. This model helped to account for the variability in light among days. This strategy could also be implemented in further analyses by including the time when the picture was taken (if more than one batch of pictures are produced) as a block term. For the validation, the other 18% of the points was used for comparing the model-predicted TAcy estimates and the real TAcy values. A total of 1000 iterations were conducted and Pearson's correlations were computed for each iteration. The mean correlation across the 1000 iterations was 0.829 (the 95% CI was 0.808 to 0.851).

Since some of the shape-related parameters are highly correlated (mean correlation among shape-related parameters = 0.705, see S1 Dataset) and some of the traits (such as volume of projected area) are not easily measurable using manual methods, validation of shape parameters was made using only length and width. Measurements for length (cm) and width (cm) of 75 cranberry fruits were manually recorded using a caliper, and pictures of these fruits were taken in batches of 25 fruits. Similar to our color validation, bootstrapping cross-validation (five-fold, 1000 iterations, 82% of the data points for model construction) was computed separately for both length and width, and mean correlations as well as confidence intervals were determined. For width, mean correlation was 0.924 (the 95% CI was 0.921 to 0.927), and for length, the mean correlation was 0.970 (95% CI was 0.969 to 0.972). Since the GiNA recognition algorithm assumes length to be the axis with the maximum length, and width to be the axis with the minimum length, length and width will be flipped in highly circular objects where the width is longer than the length. A complete dataset with all the measurements generated by GiNA as well as the manually generated data for the described analysis is available in S1 Dataset.

### Noteworthy aspects of GiNA for massive phenotyping

**Fig 2:**
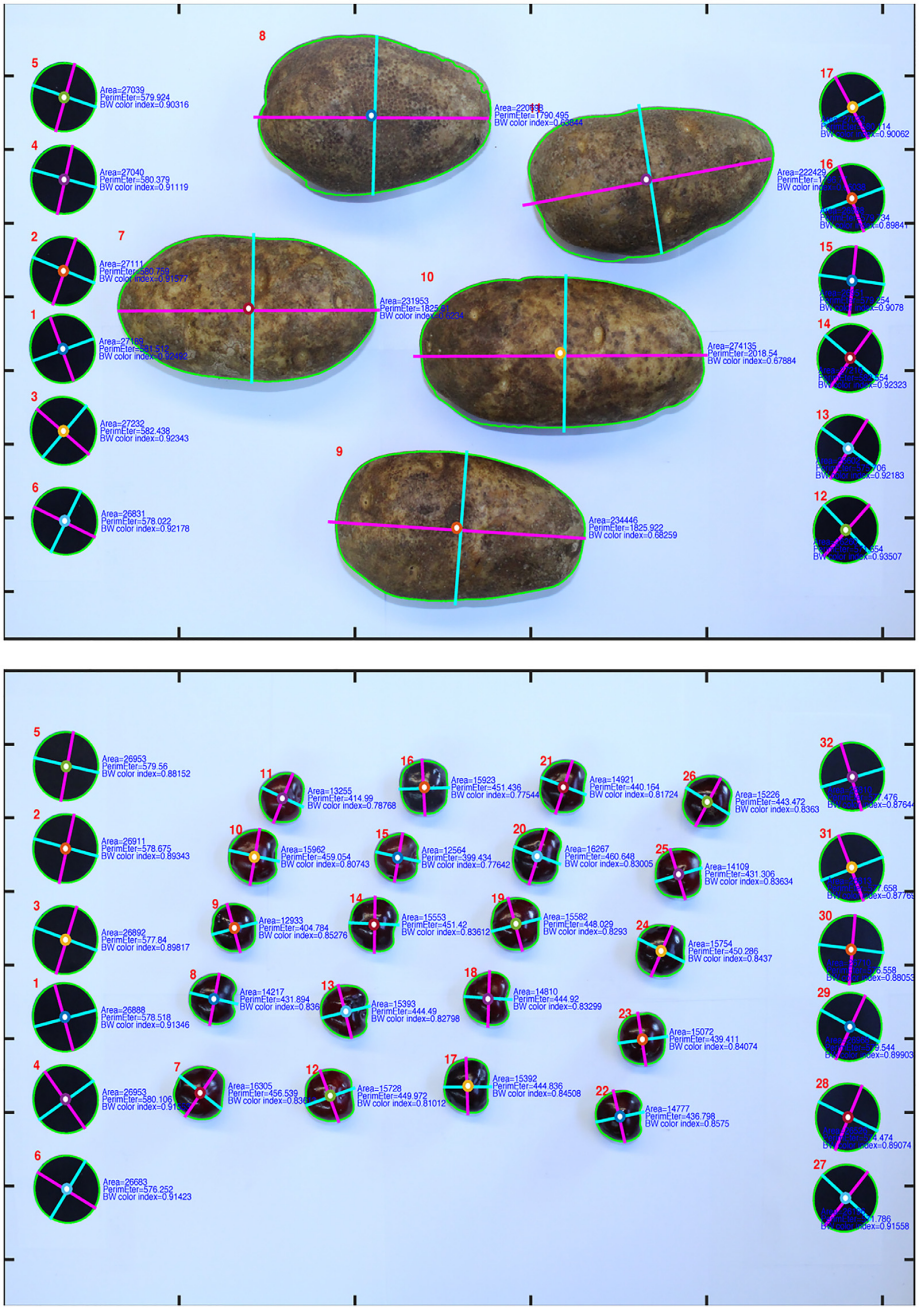
Examples of object recognition using GiNA in potato and cherry pictures. The labels in each fruit indicate preliminary parameters such as area, perimeter and gray-scale color. The lines indicate perimeter (green), length (magenta) and width (blue).

The GiNA image analysis framework is highly accessible and freely available to scientists and groups working in major and minor crop research programs (Fig 2). The application and use of this software is simple, but very helpful in terms of the massive amount of high-quality measurements that can be generated. For small fruits such as grapes, cranberries, or cherries (Fig 2), a picture of 40 fruits can be taken every minute (or less). Therefore, in an hour, at least 20 different parameters for 2400 fruits can be accurately measured from 60 images. The same amount of work would represent at least 20 man-hours to collect using traditional manual measurements. As discussed before, although many image-based phenotyping technologies are available, they are not easy-to-use and optimize, and they are not economically accessible for scientists that commonly face limitations related with massive phenotyping activities.

In crop breeding programs, massive phenotyping is key for the efficient evaluation and selection of new cultivars and varieties. In these cases, multiple populations with numerous individuals are constantly evaluated phenotypically requiring a considerable investment in time and money. The need for new approaches to acquire high-dimensional phenotypic data on an organism-wide scale will continue to increase in coming years. Although abundant genomic information is available for many plant species, as Houle *et al*. [1] commented, the characteristics of organisms of greatest interest to most biologists are phenotypes rather than genotypes. In this sense, GiNA represents an accessible and very useful tool for image-based phenomics that can lead to an accelerated progress in crop improvement and a more efficient characterization of traits of interest for both science and industry.

## Supporting Information

S1 Dataset Comparison between manual-taken measurements and GiNA estimates (75 cranberry fruits in three pictures). (XLSX)

S1 Fig Image capture device. In the figure different angles of the imaging devices is shown to highlight the simplicity required to implement this methodoloy. The device was a hand-made wooden structure with the camera set at the top of the structure facing down to capture the fruits in the white mat with black reference circles.
(TIF)

S2 Fig An example of extra images required for neural network training. In the upper panels, a representation of two colors present in fruits as well as the color of the background.
(TIF)

S3 Fig Pictures used for estimating correlation of measurements across different light conditions. (TIF)

S4 Fig Pictures used for estimating correlation of measurements in different system set ups. (TIF)

S1 Table Description of the parameters generated by GiNA. Mathematical formulation and description of parameters returned by the software.
(DOCX)
