# Supplementary figures and images for: GiNA, an Efficient and High-Throughput Software for Horticultural Phenotyping
Source: PLoS One. 2016 Aug 16;11(8):e0160439. doi: 10.1371/journal.pone.0160439 (PMC4986961; doi:10.1371/journal.pone.0160439)

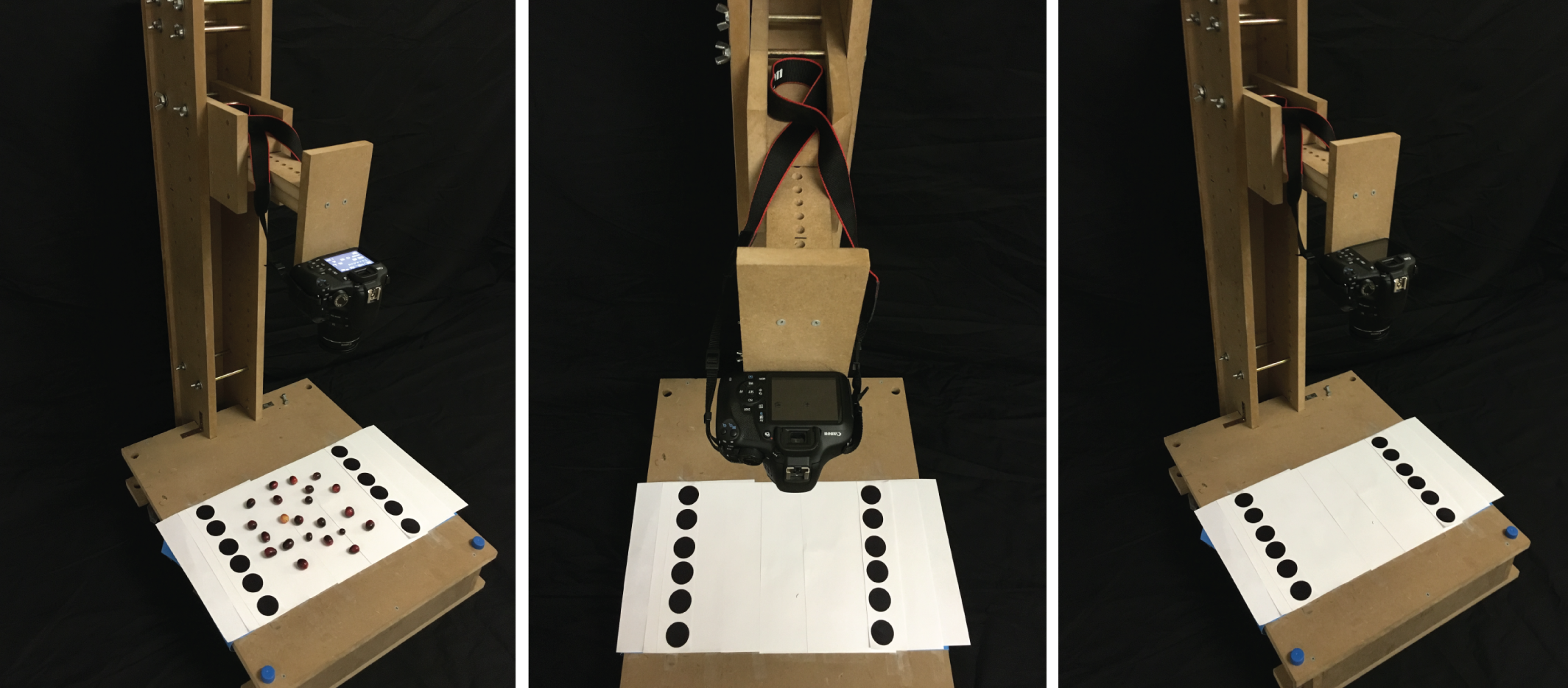

Supplement: S1 Fig — The device was a hand-made wooden structure with the camera set at the top of the structure facing down to capture the fruits in the white mat with black reference circles. (TIF) [file pone.0160439.s002.tif]

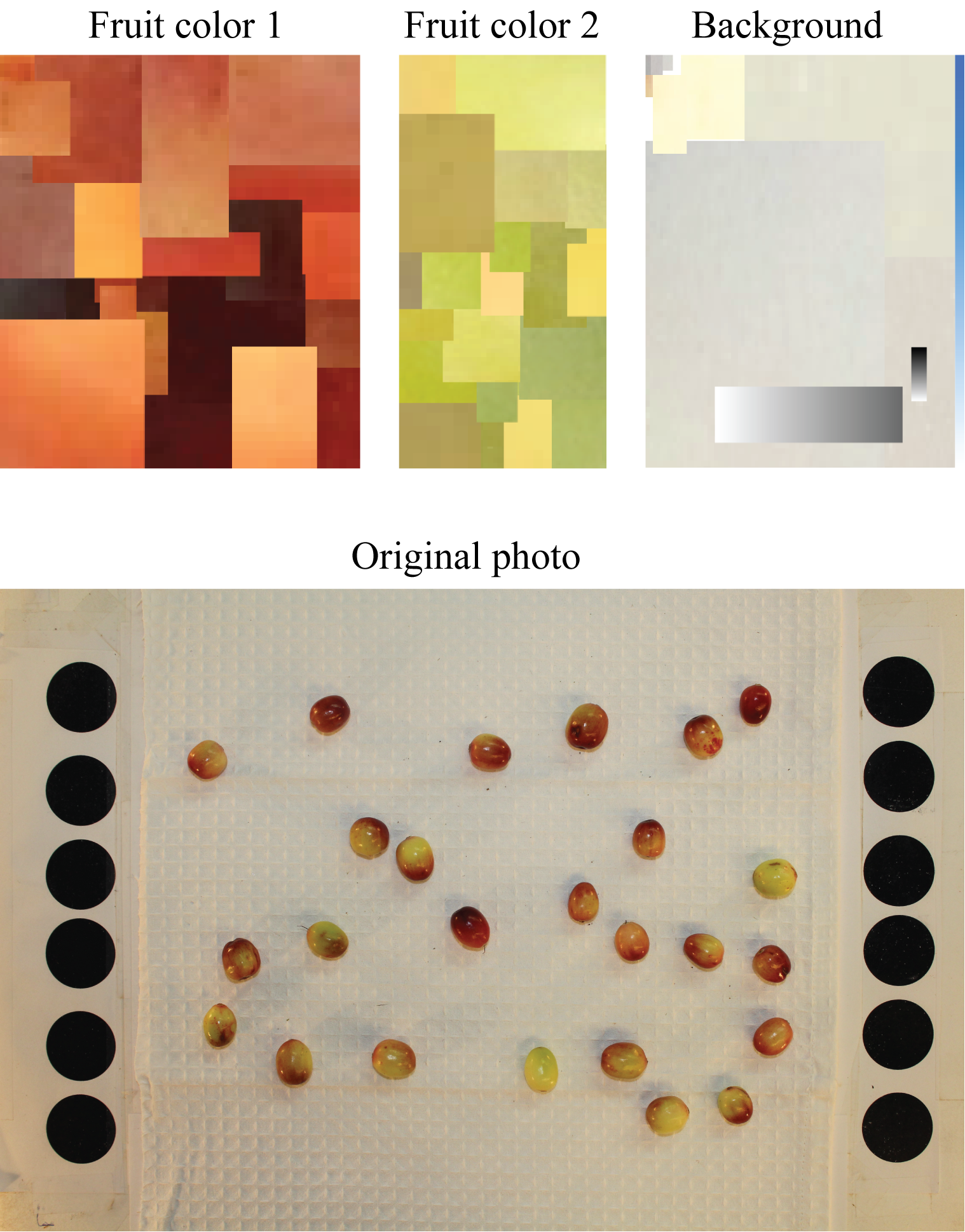

Supplement: S2 Fig — In the upper panels, a representation of two colors present in fruits as well as the color of the background. (TIF) [file pone.0160439.s003.tif]

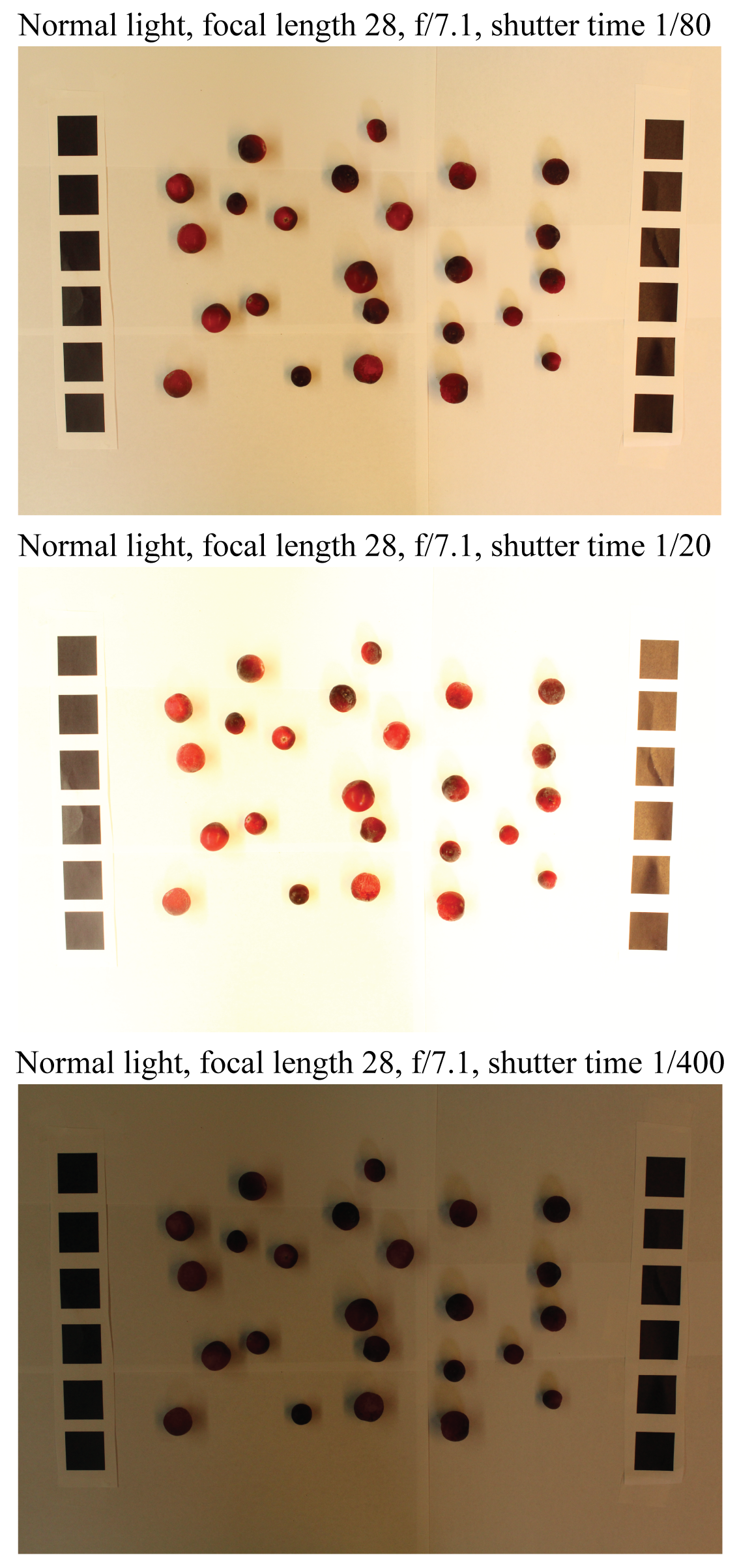

Supplement: S3 Fig — (TIF) [file pone.0160439.s004.tif]

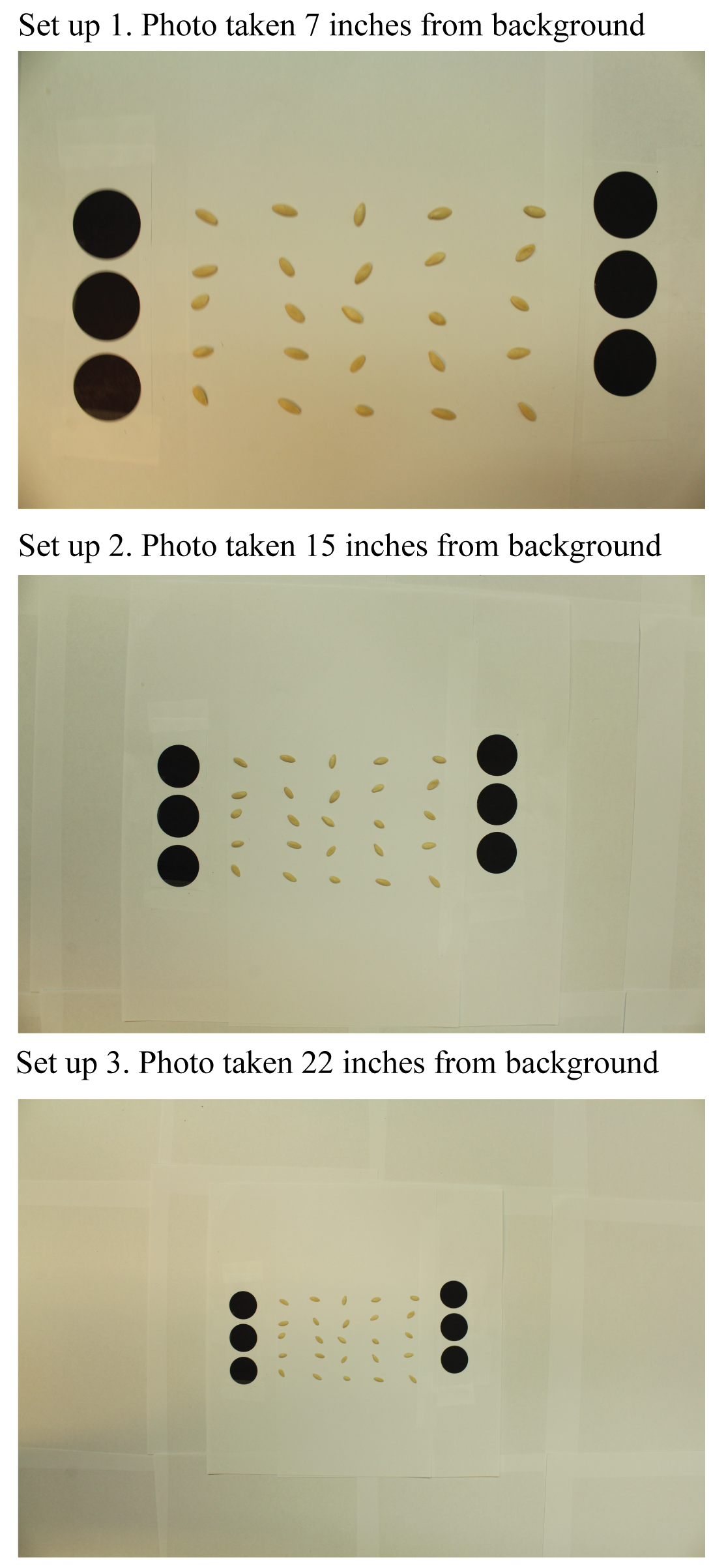

Supplement: S4 Fig — (TIF) [file pone.0160439.s005.tif]
