# Supplementary material for: GiNA, an Efficient and High-Throughput Software for Horticultural Phenotyping
Source: PLoS One. 2016 Aug 16;11(8):e0160439. doi: 10.1371/journal.pone.0160439 (PMC4986961; doi:10.1371/journal.pone.0160439)
Supplement: S1 Table — Mathematical formulation and description of parameters returned by the software. (DOCX) [file pone.0160439.s006.docx]

**Table S1. Description of the parameters generated by GiNA.** Mathematical formulation and description of parameters returned by the software.

| **Trait** | **Description** | **Formula** |
| --- | --- | --- |
| Length | Length of the major axis that has the same normalized second central moments as the region. It is always larger than the width, therefore, in objects wider than long, this parameter will be cofounded. | NA |
| Width | Length of the minor axis that has the same normalized second central moments as the region. It is always shorter than the length. | NA |
| Two-dimensional area | Number of squared units in the recognized object. The same pixels counter in this parameters are used for determine color. | NA |
| Shape | Describe how round is the object. A value of 1 represent a circle-like object. | Length / width |
| Projected skin surface | Estimation of the surface area assuming that the object is spherical or egg shaped. | $2\pi a^{2}+\mu a*2(\frac{b^{2}}{\sqrt{b^{2}-a^{2}}}\cos^{-1} \frac{a}{b})$, where $a$ and $b$ are 0.5*width and 0.5*length, respectively. |
| Two-dimensional perimeter | Distance around the boudndary region of the object. | It is computed by calculating the distance between each adjoining pair of pixels around the border of the region. |
| Projected volume | Estimation of the object volume assuming that the object is spherical or egg shaped. | $\frac{2\pi}{3}a^{2}(a+b)$, where $a$ and $b$ are 0.5*width and 0.5*length, respectively. |
| Eccentricity | Eccentricity of the ellipse that has the same second-moment as the object. An object with eccentricity of 0 is a perfect circle, while a value of 1 represent a line. | Ratio of the distance between between the foci of the ellipse and length |
| RGB-color | Average of RGB values considering all the pixels in the object. | NA |
| Gray-scale color | A transformation of the RGB to a gray-color space. | NA |
| Color variation | Variance of the RGB color and gray-scale color across all the pixels in the object. | NA |
